# Supplementary material for: The association between attachment pattern and depression severity in Thai depressed patients
Source: PLoS One. 2021 Aug 18;16(8):e0255995. doi: 10.1371/journal.pone.0255995 (PMC8372919; doi:10.1371/journal.pone.0255995)
Supplement: Attachment — Submitted filename: Rebuttal letter.docx [file pone.0255995.s002.docx]

**Rebuttal letter**

___________________________________________________________

We thank the editor and the two reviewers for their comments on our manuscript. Below is

our response to each point raised by the academic editor and reviewers. We hope that we

satisfyingly addressed them and that the manuscript will be now suited for publication.

Sincerely,

On behalf of all authors,

Chotiman Chinvararak

**Academic editor:**

1. Please ensure that your manuscript meets PLOS ONE's style requirements, including those for file naming. The PLOS ONE style templates can be found at

We have already ensured our manuscript meet the requirements.

**2. Financial disclosure**

The authors received no funding for this work. So, no party apart from authors had any role in study design, data collection and analysis, decision to publish, or preparation of the manuscript.

.

**Reviewer #1:**

**Q1: With regard to the introduction of MDD, your research can refer to some newer literature, such as “Major Depressive Disorder: Advances in Neuroscience Research and Translational Applications.Li Z, Ruan M, Chen J, Fang Y.Neurosci Bull. 2021 Feb 13”.**

We cited the data from “Major Depressive Disorder: Advances in Neuroscience Research and Translational Applications.Li Z, Ruan M, Chen J, Fang Y.Neurosci Bull. 2021 Feb 13” in the introduction on line 52.

**Q2: In the methods section, the authors doesn’t mention the method to estimate sample size. Have you estimated the sample size in your study, and what is the specific method?**
We added the method of sample size calculation on lines 65-67.

**Q3: In the results section, your work lists a lot of participant’s characteristics about the enrolled patients, but are there differences in these characteristics among people with different attachment patterns? If so, when comparing the score difference of BDI-II between different attachment patterns, it may be a better choice to use the differences in participant’s characteristics as covariates to perform a covariance analysis.**

We already did multiple logistic regression analysis adjusted for sex, education, history of medical illness, and history of psychiatric hospitalization.

**Q4: In the results section, are there some problems with the layout of Table 4?**

We adjusted the layout of Table 4 in the results section. **Q5: In the results section, if “Secure/Dismissing and Preoccupied/Fearful” layout in Table 4 means the number of Secure attachment pattern plus the number of Dismissing attachment pattern and the number of Preoccupied attachment pattern plus the number of Fearful attachment pattern and what is the basis for such a combination?**

We categorized preoccupied and fearful pattern as an anxious attachment. On the other hand, secure and dismissing patterns were categorized as a non-anxious attachment according to reference 12. Furthermore, an anxious attachment tended to have more severe depression and report their symptoms than those with a non-anxious attachment according to references 19, 27, 28 and 30. **Q6: In the results section, your research adjusted sex, education, history of medical illness and history of psychiatric hospitalization to run stepwise multiple logistic regression, what’s your reason to adjust these variables?**

We used these variables to run stepwise multiple logistic regression because they were significant factors from the theoretical review (from references 1, 4-10).

**Reviewer #2:**

**1. An AUC-ROC curve would be helpful to demonstrate the capacity of preoccupied/fearful attachment in predicting depression.**

We added the discriminatory capacity of anxious attachment style in predicting moderate to severe depression via an AUC-ROC curve on lines 126-127 and figure 1.  **2. It would be interesting to know the proportion of the participants with suicide attempts in Thai depressed patients. A pioneering study (listed below) has demonstrated that comorbid anxiety and metabolic abnormalities are also associated with severe major depressive disorders (MDD) with suicide attempts. Please include this in the discussion.**

We cited the data of comorbid anxiety and metabolic abnormalities form “The association of clinical correlates, metabolic parameters, and thyroid hormones with suicide attempts in first-episode and drug-naïve patients with major depressive disorder comorbid with anxiety: a large-scale cross-sectional study. Transl Psychiatry. 2021 Feb 4;11(1):97.” in the introduction (on lines 46-47) as well as the discussion (on lines 145-146).

We modified the manuscript to address the points made by the editor and the reviewers. We agreed with the comments in all accounts. We believe that the manuscript is now more readable, more informative, and its conclusions more useful to the public.
